# Editorial: ICF-based rehabilitation for neurological disease

**DOI:** 10.3389/fresc.2022.995070

**Published:** 2022-10-25

**Authors:** Clynton L. Correa, Tsan-Hon Liou, Maite Barrios

**Affiliations:** ^1^ Graduate Program of Physical Education, Federal University of Rio de Janeiro, Rio de Janeiro, Brazil; ^2^ Department of Physical Medicine and Rehabilitation, Taipei Medical University, Taipei, Taiwan; ^3^ Department of Social Psychology and Quantitative Psychology, University of Barcelona, Barcelona, Spain

**Keywords:** neurorehabilitation, ICF (international classification of functioning, functioning, healthcare, neurological disease

**Editorial on the Research Topic** ICF-based rehabilitation for neurological disease By Correa CL, Liou T-H and Barrios M. (2022) Front. Rehabilit. Sci. 3: 995070. doi: 10.3389/fresc.2022.995070

The International Classification of Functioning, Disability and Health (ICF) is a health model proposed and adopted by the World Health Organization (WHO) in 2001 based on a biopsychosocial model functioning and disability in any health condition (1–3).

According to the WHO, neurological diseases affect up to one billion people worldwide irrespective of age, sex, education or income. In addition, people with neurological disorders as well as their families and caregivers have difficult to access appropriate care.

People with neurological diseases have their functioning affected, can lead limit activities and/or restrict participation. The ICF is a complex interaction among the domains *Body functions*, *Body structures* and *Activities and participation* in community life. In addition, all these domains are influenced by *Environmental* and *Personal factors*. Based on this fact, approaching to disability needs multidisciplinary intervention (4). In this sense, rehabilitation is one of the key components to be offered by patients suffering neurological diseases. Rehabilitation is offered to people with diverse health conditions affected by diseases in order to reach recovery in different aspects (physical, mental and social) into their environment (5). The rehabilitation process must be considered as an integrative model based on rehabilitation strategy which contains four steps namely: Assessment, Assignment, Intervention, and Evaluation (4).

Taking together ICF and rehabilitation, persons suffering neurological diseases can be positively impacted by health professionals adopting the biopsychosocial model. The aim of this Research Topic is to provide readers with information on ICF aspects including, but not limited to, ICF-based tools for use with people with neurological disease in different areas.

The Research Topic contains four articles focusing on different life-cycles from infants to adults. Besides, we introduce our readers to works using the ICF in different Rehabilitation scenarios. Formiga et al. performed a case report describing the health history and development based on ICF domains of a high-risk preterm infant born to a mother hospitalized due to COVID-19 complications Formiga et al. (2022). Lima et al. present the increase in ICF activity domain in hospitalized neurological patients. Lindner and Buer carried out study based on protocol for neurological diseases. These authors linked rehabilitation goals to the ICF domains focusing on assistive technology for cognition Lindner et al. (2022). Finally, Capato et al. present a case report of a woman with Huntington's disease and with severe chorea medication-refractory that multidisciplinary was assessed based on ICF domains in both home and clinical settings to study the disability, including contextual factor before and after surgery. Their results show how ICF-based evaluations were useful to identify the efficacy of the long-term of multidisciplinary intervention and guide clinician's decisions Capato et al. (2022).

We hope these papers can stimulate a critical thinking in our readers to use the ICF model in different Rehabilitation clinical settings.
